# ArgT functions as an arginine transporter in *Staphylococcus aureus*

**DOI:** 10.1128/jb.00362-25

**Published:** 2025-11-28

**Authors:** Gabrielle F. Schulze, Itidal Reslane, Fareha Razvi, Luke D. Handke, McKenzie K. Lehman, Paul D. Fey

**Affiliations:** 1 Department of Pathology, Microbiology, and Immunology, Center for Staphylococcal Research, University of Nebraska Medical Center 12284, Omaha, Nebraska, USA; The Ohio State University, Columbus, Ohio, USA

**Keywords:** arginine, carbon catabolite repression, amino acid transporters, *Staphylococcus aureus*

## Abstract

Glycolytic activity is required for *Staphylococcus aureus* (*S. aureus*) to establish an infection. These data indicate that carbon catabolite repression, mediated by CcpA, is active during the initial stages of infection. CcpA represses both the biosynthesis and catabolism of arginine; therefore, it is hypothesized that arginine must be transported by *S. aureus* from host tissue to facilitate growth during the establishment of an infection. Within *S. aureus* USA300, two known arginine/ornithine antiporters, ArcD1 and ArcD2, are encoded on the two copies of the arginine deiminase operon (native and arginine catabolite mobile genetic element-derived). However, when both antiporters are inactivated via allelic replacement, no growth defect is observed in defined medium where arginine is required for growth, indicating that *S. aureus* contains additional arginine transporters. Using the toxic arginine analog, canavanine, we identified a novel *S. aureus* arginine transporter, SAUSA300_*2383* (Arginine Transporter; ArgT). Transcriptional analysis found that *argT* was regulated by both the canonical arginine biosynthesis repressor AhrC and CcpA; thus, its transcription is repressed during growth in medium containing glucose and is therefore not the primary arginine transporter during growth in medium containing glucose. However, we found that growth is dependent upon ArgT during growth in medium lacking proline, which suggests that *S. aureus* has evolved a specific response to accommodate proline-depleted growth conditions.

**IMPORTANCE:**

*Staphylococcus aureus* is a leading cause of both community and hospital-acquired infection worldwide. In addition, *S. aureus* is resistant to many commonly used antibiotics, which make the treatment of bacteremia, infective endocarditis, and other invasive diseases more challenging. It is essential to obtain a basic understanding of how *S. aureus* survives in a variety of host niches, including those niches where *S. aureus* is dependent upon amino acid catabolism. We hypothesize that arginine acquisition is critical for *S. aureus* pathogenesis; therefore, identifying these transporters is essential for the development of novel therapeutic strategies.

## INTRODUCTION

*Staphylococcus aureus* (*S. aureus*) colonizes approximately 30% of the population and can cause metastatic disease typically following a primary infection (1–3). To establish an infection, Vitko, Spahich, and Richardson and Somerville and Proctor (4, 5) have shown that *S. aureus* requires glucose as a carbon and energy source. Therefore, during the establishment of an infection, glucose-mediated carbon catabolite repression (CCR) via CcpA is active. CcpA is known to repress several genes that function in the biosynthesis of arginine using proline as a substrate, including *putA*, *rocD*, *arcB1*, and *argGH* (6, 7). Indeed, growth in media containing glucose but lacking arginine is dependent upon the absence of CcpA, thereby de-repressing arginine biosynthesis (6). Consequently, we hypothesize, in the presence of glucose, *S. aureus* is dependent on arginine transport for survival.

In *S. aureus*, arginine is used as a substrate for several metabolic reactions outside of its function as a proteinogenic amino acid. First, arginine is a substrate for bacterial nitric oxide synthase that functions to generate NO^•^ and eventually NO_2_ thus stimulating aerobic respiration (8). In addition, arginase (RocF) utilizes arginine as a substrate generating ornithine and urea (9, 10). Urea can then be used to facilitate pH homeostasis via urease activity, whereas ornithine is converted to glutamate via RocD and RocA, which functions as a major carbon source in the absence of glucose (9). Importantly, arginine is also catabolized via the arginine deiminase (ADI) pathway, generating ornithine, ATP, CO_2_, and NH_3_ (10). The ADI pathway is conserved within many gram-positive bacteria and is typically utilized to produce ATP in anaerobic environments and, in addition, produce NH_3_ facilitating pH homeostasis in acidic niches of the host (10, 11). In addition, the highly conserved arginine/ornithine antiporter (*arcD1*) is encoded within the ADI operon (12, 13). Interestingly, many *S. aureus* USA300 (ST8) isolates also encode a second arginine/ornithine transporter, *arcD2*, encoded on the arginine catabolite mobile genetic element (ACME) pathogenicity island (14). The ACME-encoded ADI operon has been shown to help facilitate growth and survival within acidic environments such as the skin (14). Importantly, no known arginine transporters have been identified in *S. aureus* besides ArcD1 and ArcD2.

Arginine is a semi-essential amino acid in humans and, along with its precursors glutamine, glutamate, and proline, is acquired from the diet at a rate of 3–5 g/day (15–17). When arginine is ingested, the small intestine absorbs approximately 40%, while the remaining 60% is utilized for protein synthesis, absorbed by the liver for subsequent urea production, or secreted into the bloodstream (50–150 µM) (16). Further, during an immune response, inducible nitric oxide synthase uses arginine as a substrate to synthesize NO**∙** and functions in both the adaptive and innate immune host inflammatory defense (15, 16). Thus, during staphylococcal metastasis via the vasculature and subsequent tissue invasion, both niches where glucose is abundant, we postulate that arginine is available for transport.

In this study, we identified an arginine transporter, ArgT, that is required for growth in chemically defined medium (CDM) lacking proline. Transcription of *argT* is regulated by both CcpA and the canonical arginine-responsive regulator AhrC. However, ArgT is not required for growth in media containing glucose, suggesting that other arginine transporters remain to be identified in *S. aureus*.

## RESULTS

### *S. aureus* encodes unidentified arginine transporters

**Fig 1:**
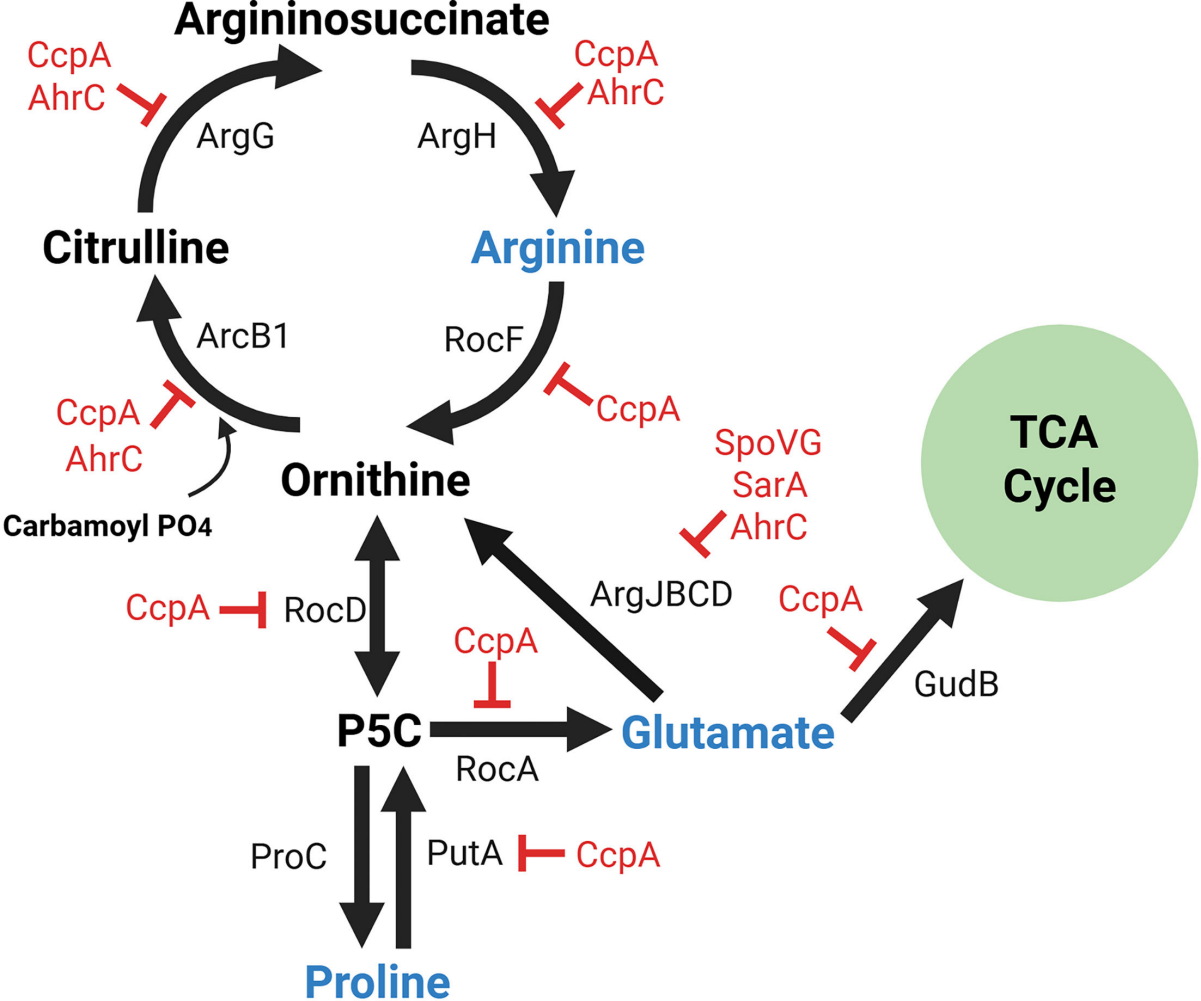
Regulation of *S. aureus* arginine metabolism. In CDM, *S. aureus* is an arginine auxotroph due to transcriptional repression of anabolic pathways. *S. aureus* encodes pathways to synthesize arginine from proline and glutamate; however, these pathways are repressed by regulatory proteins (AhrC, SarA, and SpoVG). Therefore, in a medium lacking glucose, proline, and glutamate (CDM-PE), growth is dependent on the acquisition of exogenous arginine. P5C = pyrroline-5-carboxylate. Made with BioRender.

**Fig 2:**
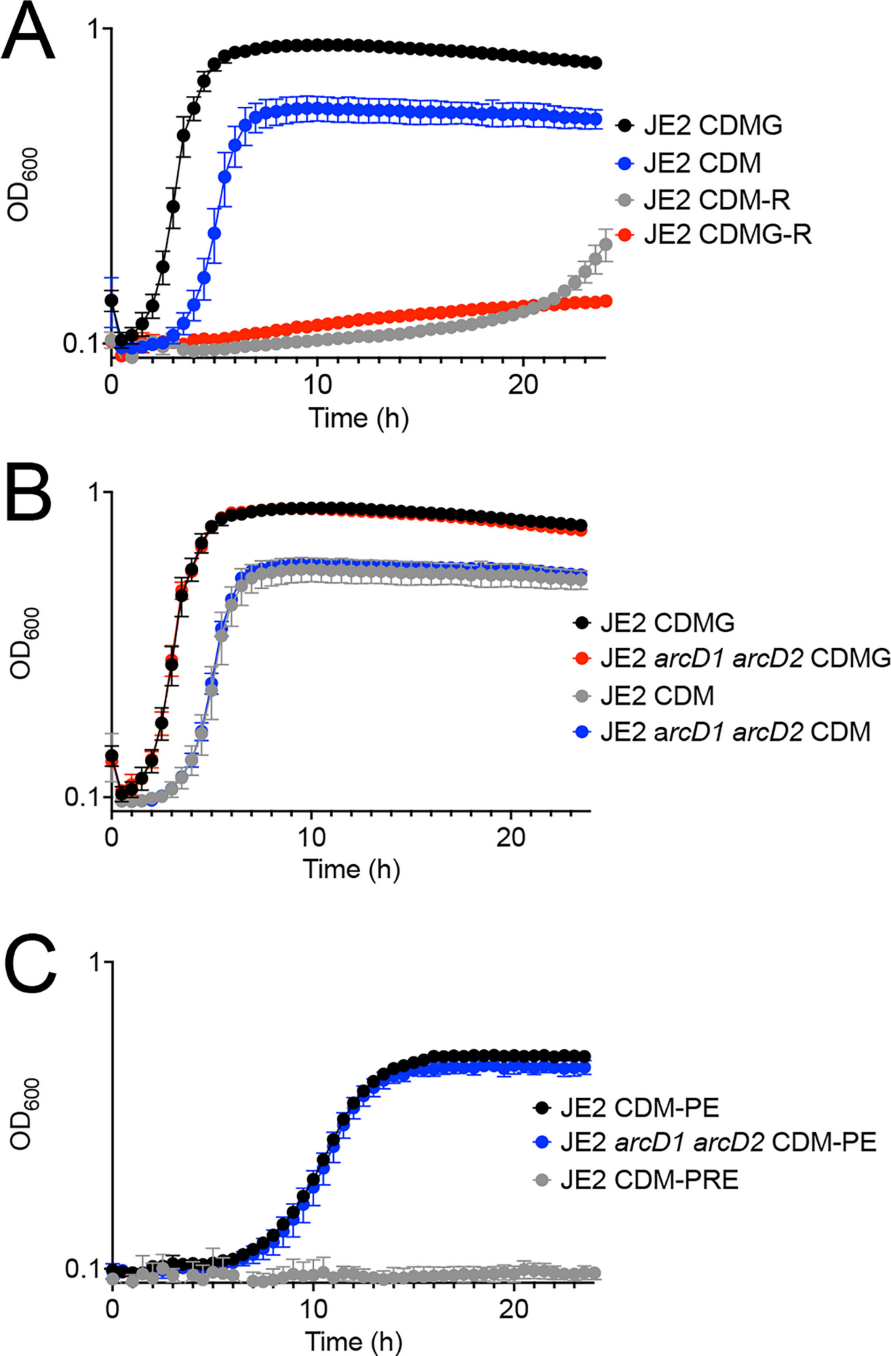
*S. aureus* encodes unidentified arginine transporters. (**A**) Growth analysis of JE2 in CDM and CDMG with and without arginine (CDMG-R and CDM-R). (**B**) Growth analysis of JE2 and JE2 *arcD1 arcD2* grown in CDM and CDMG or (**C**) CDM-PE or CDM-PRE. Data are represented as mean +/− SD (*n* = 3).

*S. aureus* encodes pathways to synthesize arginine from proline and glutamate; however, these pathways are repressed via AhrC, CcpA, SpoVG, and SarA, resulting in phenotypic arginine auxotrophy and thus a requirement to import arginine (7, 18, 19) (Fig. 1). This characteristic is demonstrated in Fig. 2A where JE2 exhibits robust growth in CDM with glucose (CDMG) and without glucose (CDM). However, once arginine is removed from the medium (CDMG-R/CDM-R), due to the reliance on arginine transport, growth of JE2 is abrogated. *S. aureus* USA300 isolates encode two known arginine transporters within the two ADI operons, ArcD1 and ArcD2 (12, 14, 20). To determine if ArcD1 or ArcD2 function to transport arginine for carbon acquisition, JE2 *arcD1::tet arcD2::spec* (hereafter referred to as JE2 *arcD1 arcD2*) was grown in CDM and CDMG. No impairment of growth, as compared to JE2, was noted, indicating that arginine is transported via other unidentified transporters (Fig. 2B). Growth of JE2 *arcD1 arcD2* was further tested in CDM lacking proline and glutamate (CDM-PE) where arginine functions as a critical carbon source to fuel glutamate/glutamine and proline synthesis (see Fig. 1) (7). As noted in Fig. 2C, growth of JE2 *arcD1 arcD2* grows similarly to JE2, further indicating the presence and expression of an unidentified arginine transporter(s). Collectively, these data suggest that ArcD1 and ArcD2 are not the primary arginine transporters under the conditions tested and presumably function primarily during ADI induction (14, 20). Further, as ArcD1 and ArcD2 are thought to be arginine/ornithine antiporters, their use for carbon acquisition would represent a futile cycle as ornithine would be required to serve as a substrate to yield pyrroline-5-carboxylate and glutamate (Fig. 1).

### Use of canavanine to identify arginine transporters

**Fig 3:**
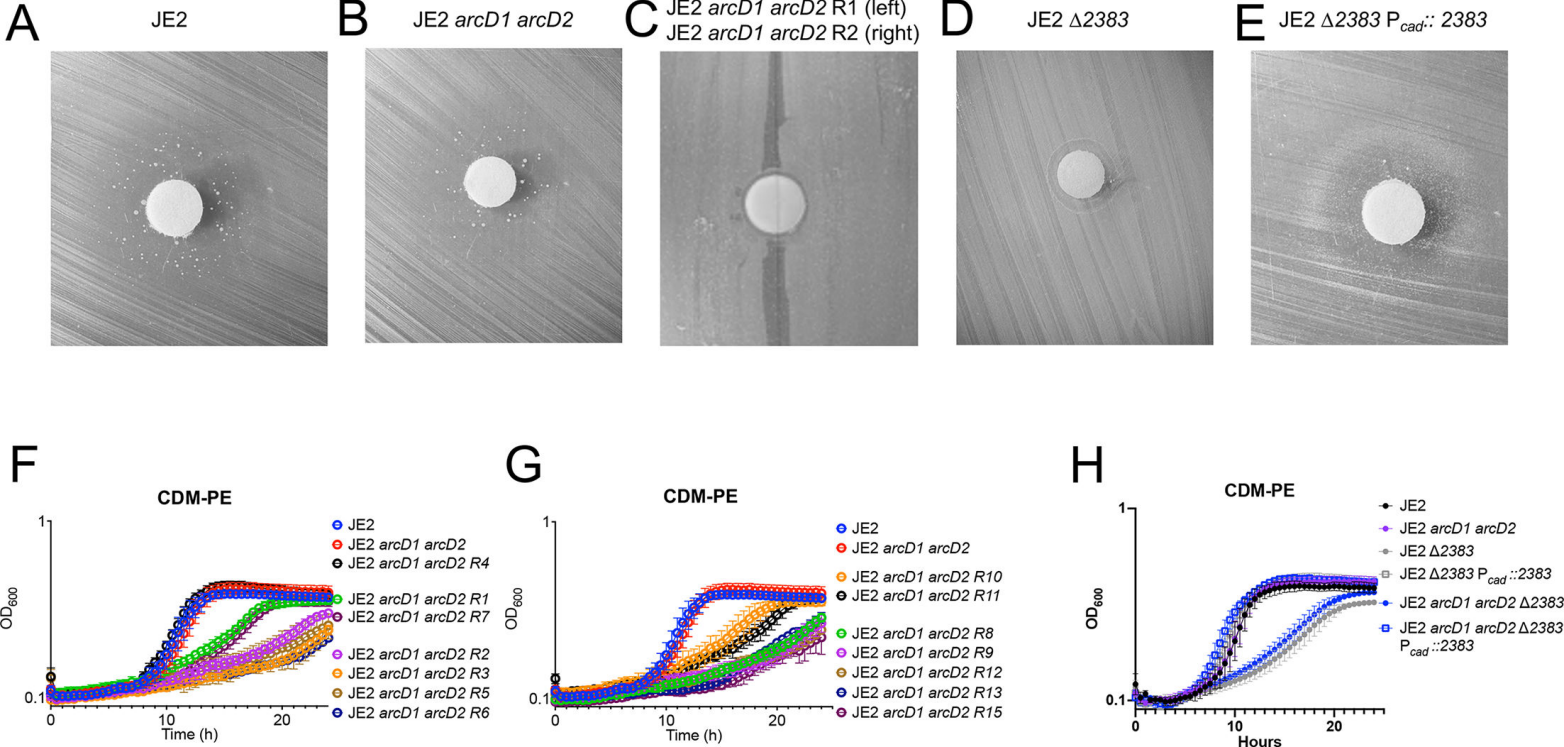
The identification of a novel *S. aureus* arginine transporter, SAUSA300_2383. A lawn of JE2 (**A**) or JE2 *arcD1 arcD2* (**B**) was grown on CDM agarose plates lacking proline, glutamate, and arginine with the addition of 0.1% trypticase peptones. The toxic arginine analog, canavanine, was added to the center disk and the bacteria formed a zone of resistance. (**C**) Single colonies (suppressor mutants R1 and R2) were isolated, and their resistance against canavanine was verified. (**D**) JE2 ∆*2383* was confirmed as resistant to canavanine, and (**E**) the zone of inhibition was complemented via introduction of SAUSA300_*2383*. (**F and G**) Canavanine suppressor mutants were analyzed via growth analysis in CDM-PE where arginine transport is required for growth. All mutants, apart from R4, exhibited reduced growth compared to the parental strain. (**H**) Growth analysis of JE2, JE2 *arcD1 arcD2,* JE2 ∆*2383*, and JE2 *arcD1 arcD2 2383* and their complemented strains in CDM-PE. A disruption in *2383* impairs growth in CDM-PE; however, there is no additional growth defect exhibited with disruption of *arcD1* and *arcD2* in a ∆*2383* background. Data (**F–H**) are represented by the mean +/− SD (*n* = 3).

To identify other arginine transporters, we first utilized the toxic arginine analog canavanine to select for mutants resistant to the compound. Canavanine, once transported into bacteria via arginine transporters, causes toxicity by incorporation into protein (21, 22) where the less basic structure of canavanine causes the accumulation of misfolded proteins leading to bacterial cell death (21, 23). To isolate resistant colonies, a lawn of JE2 *arcD1 arcD2* was grown on CDM agarose lacking proline, arginine, and glutamate (CDM-PRE) containing 0.1% trypticase peptone. Since arginine is required for growth, trypticase peptone was added to serve as an arginine source in an oligopeptide permease-dependent manner (24). Canavanine was added to a sterile paper disk at the center of the plate, resulting in a zone of inhibition, indicating the toxicity of the compound (Fig. 3A and B). However, multiple large colonies were found within the zone of inhibition, suggesting that a mutation was selected facilitating either lack of canavanine transport or, potentially, detoxification of canavanine. Suppressor mutants were restreaked on plates to verify their resistance to canavanine (Fig. 3C). Growth kinetics from 14 suppressor mutants isolated from individual biological experiments were assessed in CDM-PE, where arginine serves as a primary carbon source (Fig. 3F and G). All suppressor mutants, except for JE2 *arcD1 arcD2 R4*, exhibited a decreased growth rate compared to the parental strain and were therefore subsequently sequenced. Whole genome sequencing found that 13 isolates contained unique single nucleotide polymorphisms (SNPs), resulting in amino acid changes, in SAUSA300_*2383* (*S. aureus* USA300_FPR3757 annotation) encoding a putative amino acid transporter (Table 1). Bioinformatic analysis, via InterPro, of SAUSA300_*2383* revealed a SLC12A domain, indicating the protein contains 12 transmembrane domains involved in the coupled transport of sodium and/or potassium (25, 26). The permease belongs to the APC superfamily, which encodes for membrane-bound permeases that are involved in the active transport of amino acids (27).

**TABLE 1:** Whole genome sequencing of canavanine-resistant strains^*a*^

Strain	Amino acid change	Locus (*S. aureus* FPR3757)	Protein encoded
R1	Ile → Asn (275) Ser → Leu (70)	SAUSA300_*2383* SAUSA300_*1731*	Amino acid permease Phosphoenolpyruvate carboxykinase
R2	Deletion (227) Asp → Gly (454) Lys → Ile (202)	SAUSA300_*2383* SAUSA300_*0222* SAUSA300_*2126*	Amino acid permease Glycerophosphodiester phosphodiesterase MFS transporter
R3	Ser → Thr (294) Val → Phe	SAUSA300_*2383* SAUSA300_*1639*	Amino acid permease DNA-binding response regulator
R4	Thr → Ile (233) Gln → Leu (327) Met → Ile (155) Val → Ile (16) Arg → Leu (154)	SAUSA300_*0085* SAUSA300_*0869* SAUSA300_*1470* SAUSA300_*1607* SAUSA300_*2438*	Dihydroneopterin aldose ATP dependent helicase Geranyl transferase Hypothetical protein Transcriptional regulator
R5	Arg → Pro (305) Lys → Glu (95)	SAUSA300_*2383* SAUSA300_*0830*	Amino acid permease Hypothetical Protein
R6	Gly → Asp (165) Deletion (18)	SAUSA300_*2383* SAUSA300_*1010*	Amino acid permease DUF2197 domain-containing protein
R7	Ala → Asp (378) Deletion (20) Glu → Val (23) Leu → Gln (436)	SAUSA300_*2383* SAUSA300_*0327* SAUSA300_*2152* SAUSA300_*2167*	Amino acid permease Deacetylase SIR2 Tagatose 1,6-diphosphate aldolase Hypothetical protein
R8	Arg → Pro (305)	SAUSA300_*2383*	Amino acid permease
R9	Ala → Val (295)	SAUSA300_*2383*	Amino acid permease
R10	Ala → Asp (291)	SAUSA300_*2383*	Amino acid permease
R11	Asn → Lys (184) His → Asp (649)	SAUSA300_*2383* SAUSA300_*0238*	Amino acid permease Transcriptional anti-terminator
R12	Deletion (24-26) Phe → Leu (28) Leu → Ile (29) Deletion (31-40) Ser → Cys (41)	SAUSA300_*2383* SAUSA300_*2383* SAUSA300_*2383* SAUSA300_*2383* SAUSA300_*2383*	Amino acid permease
R13	Leu → Pro (42)	SAUSA300_*2383*	Amino acid permease
R15	Gly → Arg (26)	SAUSA300_*2383*	Amino acid permease

^*a*^ Compared to the *S. aureus* USA300 reference genome FPR3757.

To verify the resistance to canavanine is due to inactivation of the SAUSA300_*2383* gene, a SAUSA300_*2383* allelic replacement mutant was constructed. JE2 ∆*2383* was grown on CDM-PRE plates (containing trypticase peptones) in the presence of canavanine and compared to the parental suppressor strain (Fig. 3D). Indeed, JE2 ∆*2383* was able to grow in the presence of canavanine, indicating that a mutation in the SAUSA300_2383 gene led to canavanine resistance. Introduction of SAUSA300_*2383* using a non-cognate promoter (P*_cad_*) restored susceptibility to canavanine (Fig. 3E). Therefore, the SAUSA300_*2383 Tn* insertion mutation from the Nebraska Transposon Mutant Library (NTML) was transduced via Φ11 into JE2 *arcD1 arcD2*, creating JE2 *arcD1::tet arcD2::spec 2383::erm* (hereafter noted as JE2 *arcD1 arcD2 2383*) and subsequently grown in CDM-PE (Fig. 3H). Growth of JE2 *arcD1 arcD2 2383* and JE2 ∆*2383* in CDM-PE was markedly reduced as compared to JE2, suggesting that SAUSA300_*2383* may function as an arginine transporter during growth in medium lacking glucose and where arginine serves as a primary proline and glutamate source. Notably, SAUSA300*_2383* is upstream of SAUSA300_*2384*, encoding a putative Na+/H+ antiporter. To determine if SAUSA300_*2383* and SAUSA300_*2384* function in concert to import arginine, both JE2 ∆*2383* and JE2 *2384::Tn* were grown in CDM-PE. Growth of JE2 *2384::Tn* phenocopied JE2 in this medium, in comparison to the reduced growth rate and yield of JE2 ∆*2383*, suggesting that SAUSA300_*2383* and SAUSA3000_*2384* do not function together under the conditions tested (Fig. S1).

### Unidentified transporters function to transport arginine in medium replete with proline and glutamate

**Fig 4:**
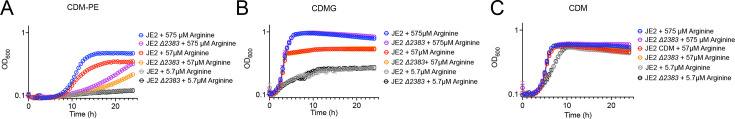
JE2 ∆2383 exhibits reduced growth in CDM-PE with low arginine concentrations. Growth analysis of JE2 and JE2 *∆2383* in CDM-PE (**A**), CDMG (**B**), and CDM (**C**). Strains were grown in either 575 µM arginine and reduced arginine concentrations of 57 and 5.7 µM. JE2 *∆2383* exhibited reduced growth exclusively in CDM-PE conditions, with growth being further reduced when arginine concentration was lowered to 57 µM. Data (**A–C**) are represented by the mean +/− SD (*n* = 3).

**Fig 5:**
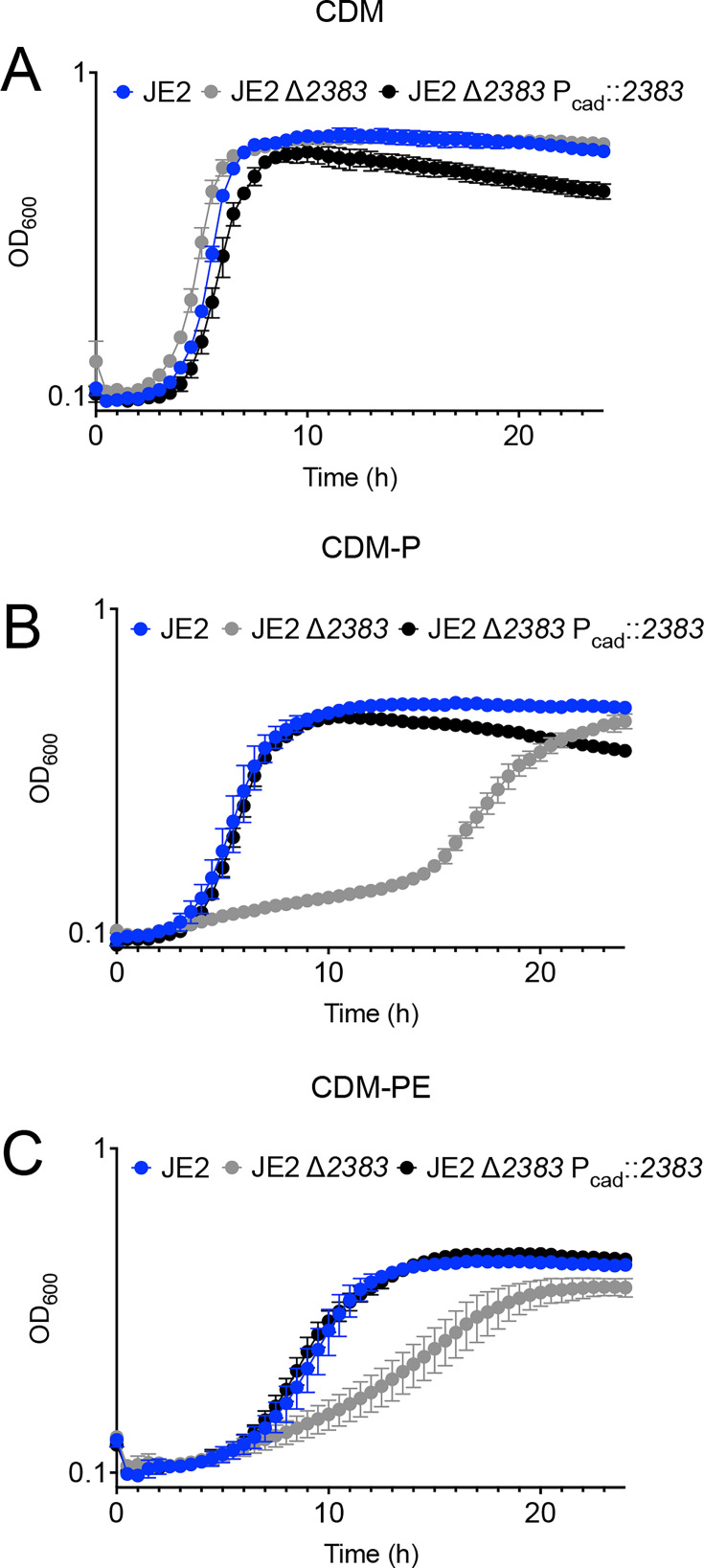
*SAUSA300_2383* complementation. SAUSA300*_2383* was overexpressed in JE2 ∆*2383* (JE2 ∆*2383* P_cad_::*2383*) and grown in CDM containing 100 mM cadmium chloride (**A**), CDM-P (**B**), and CDM-PE (**C**). Note the growth rescue of complemented JE2 ∆*2383* during growth in both CDM-P and CDM-PE. Data are represented by the mean +/– SD (*n* = 3).

Although the growth rate of JE2 *arcD1 arcD2 2383* is significantly reduced relative to JE2, the growth yield in CDM-PE after 24 hours is similar to JE2 (Fig. 3H), suggesting that other arginine transporters are expressed in this strain background. No growth phenotype was observed with the introduction of the *arcD1 arcD2* mutations in any of the growth medium conditions tested, further suggesting that ArcD1 or ArcD2 do not function as main arginine transporters for acquisition of carbon (Fig. S2). The concentration of arginine in CDM is 575 µM, much higher than the concentration of arginine in serum (50–150 µM) (16, 28). Since amino acid transporters can transport other amino acids in a non-specific manner (29), the concentration of arginine was reduced in CDM to physiological levels. We subsequently grew JE2 and JE2 ∆*2383* in CDM-PE containing reduced concentrations of arginine (57 and 5.7 µM). Indeed, when JE2 was grown in CDM containing 57 µM arginine, growth yield was further dependent upon SAUSA300_*2383* (Fig. 4A). However, surprisingly, when proline and glutamate were added to both CDM and CDMG, growth was not SAUSA300_*2383*-dependent, suggesting that other arginine transporters are expressed during growth in these media (Fig. 4B and C). Collectively, these data suggest that SAUSA300_2383 functions to transport arginine in proline and/or glutamate-depleted conditions. To verify if the growth defect observed from JE2 ∆*2383* is exclusively a result of a deletion in SAUSA300*_2383*, *SAUSA300_2383* was cloned into pBK123, and transcription was driven by an inducible cadmium promoter. This construct was transduced via Φ11 into JE2 ∆*2383* (∆*2383* P*_cad_:2383*) (Fig. 5). We found that overexpression of SAUSA300_*2383* rescued growth of JE2 ∆*2383* in both CDM-P and CDM-PE, while all strains grew similarly in CDM (Fig. 5A through C).

### Growth in proline-depleted media is dependent upon SAUSA300_2383

**Fig 6:**
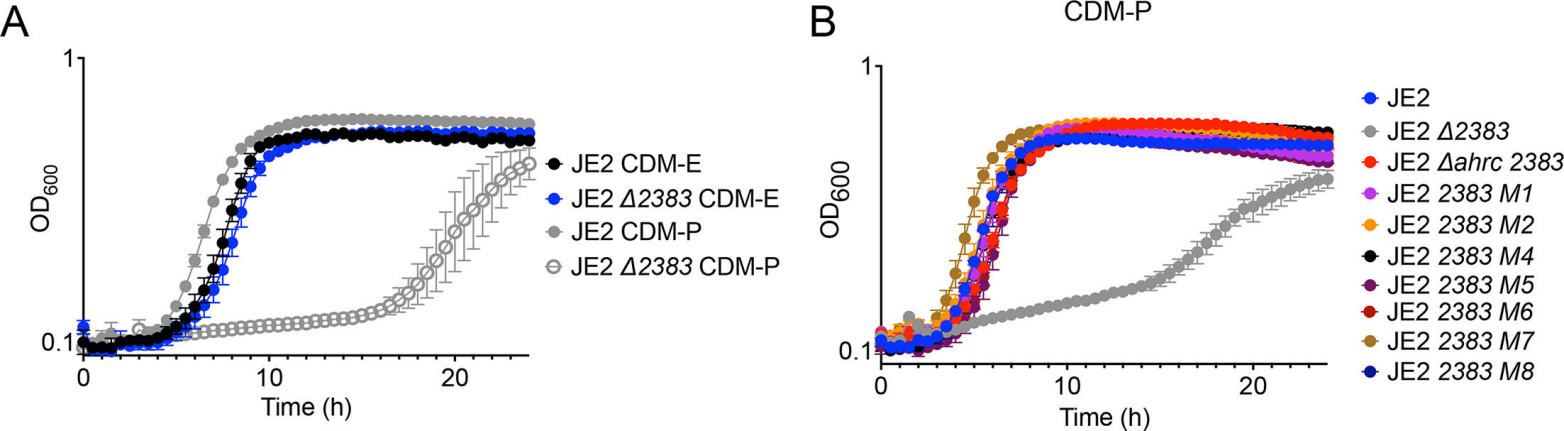
Growth in medium lacking proline is dependent upon *SAUSA300_2383*. JE2 and JE2 ∆*2383* were grown in CDM-E or CDM-P (**A**). Note that growth in CDM-P is dependent upon *SAUSA300_2383*. (**B**). Growth of JE2, JE2 ∆*2383,* JE2 ∆*ahrC,* and suppressor mutants in CDM-P. Suppressor mutants JE2 ∆*2383* M1–M8 displayed robust growth as compared to JE2 ∆*2383* due to an acquired mutation in *ahrC*. Data (**A and B**) are represented by the mean +/– SD (*n* = 3).

To determine if the dependence upon SAUSA300_*2383* was linked to either proline, glutamate, or both amino acids, *S. aureus* JE2 and JE2 ∆*2383* were grown in CDM lacking glutamate (CDM-E) or proline (CDM-P). We found that growth of JE2 ∆*2383* was not affected in CDM-E, whereas growth in medium lacking proline (CDM-P) was significantly reduced (Fig. 6A). Interestingly, as noted in Fig. 5B and 6B, growth of JE2 ∆*2383* in CDM-P is observed after 18 hours of growth. To determine if this growth phenotype is caused via the selection of compensatory mutations or due to the late induction of specific arginine transporters, JE2 ∆*2383* was plated onto TSA following 24 hours of growth in CDM-P, and single colonies were isolated. Eight colonies from independent biological experiments were then regrown in CDM-P (Fig. 6B). As noted in Fig. 6B, the eight individual JE2 ∆*2383* colonies (M1–M8) grew robustly in CDM-P. These data suggest that compensatory mutations allowed for growth in CDM-P. Strains that acquired the ability to grow were analyzed via whole-genome sequencing to identify the mutation responsible for restoring growth. Notably, five of the eight strains that phenocopied wild type had acquired a mutation in *ahrC*, an arginine transcriptional regulator belonging to the ArgR family of regulators (Table 2) (18, 19, 30). To confirm the impact of an *ahrC* mutation in the context of growth in CDM-P, a JE2 ∆*ahrC 2383::Tn* mutant strain was constructed. Indeed, an *ahrC* mutation facilitated growth of JE2 ∆*2383* in CDM-P, suggesting that AhrC functions to repress other unidentified arginine transporters during growth in CDM-P (Fig. 6B).

**TABLE 2:** Whole genome sequencing of isolates with enhanced growth in CDM-P

Sample	Amino acid change	Locus	Protein encoded
M1	Gln →Stop (105)	SAUSA300_*1469*	AhrC
M2	Pro →Thr (99)	SAUSA300_*1469*	AhrC
M4	Ala →Val (111) Ile →Asn (392)	SAUSA300_*1469* SAUSA300_*1267*	AhrC Tryptophan synthase subunit beta
M5	Ile →Asn (392)	SAUSA300_*1267*	Tryptophan synthase subunit beta
M6	Ile →Asn (392)	SAUSA300_*1267*	Tryptophan synthase subunit beta
M7	Ser →Lys (104) Ile →Asn (392) Ala →Thr (48)	SAUSA300_*1469* SAUSA300_*1267* SAUSA300_*1336*	AhrC Tryptophan synthase subunit beta RNA methyltransferase
M8	Ala →Thr (111)	SAUSA300_*1469*	AhrC

### SAUSA300_*2383* is regulated at the transcriptional level by CcpA and AhrC

**Fig 7:**
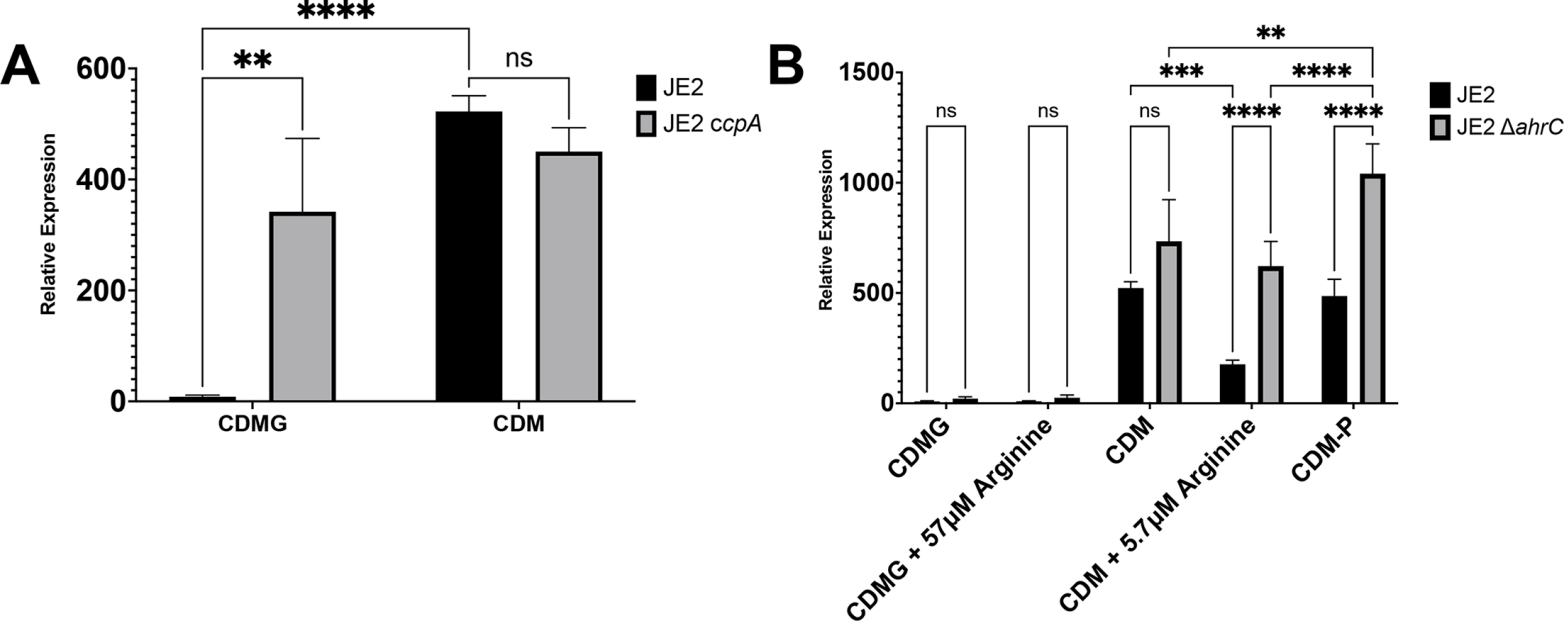
*SAUSA300_2383* is transcriptionally egulated by CcpA and AhrC. SAUSA300_*2383* transcript levels were evaluated via RT-PCR in JE2 and JE2 *ccpA* grown in CDM or CDMG with different levels of arginine (**A**). Note the lack of expression of *SAUSA300_2383* in CDMG that is dependent upon CcpA repression. (**B**) Assessment of SAUSA300_*2383* transcript following growth of JE2 and JE2 ∆*ahrC* in CDMG (574 µM arginine), CDMG with 57 µM arginine, CDM (574 µM arginine), CDM with 5.7 µM arginine, and CDM-P. Note AhrC-dependent transcript increases following growth in CDM with 5.7 µM arginine and CDM-P. Results are from three independent biological replicates with two technical replicates for each experiment. Statistical significance was assessed via a two-way analysis of variance followed by Tukey test. **, *P* < 0.01; ***, *P* < 0.001; ****, *P* < 0.0001.

As growth of JE2 ∆*2383* displayed no growth phenotype in CDM or CDMG as compared to JE2, we sought to determine the transcriptional regulation of SAUSA300_*2383*. We found that SAUSA300*_2383* was significantly higher in CDM compared to CDMG, suggesting that SAUSA300_*2383* is regulated by the CCR protein, CcpA (Fig. 7A). Indeed, SAUSA300_*2383* transcription was significantly increased when JE2 *ccpA::tet* was grown in CDMG (Fig. 7A), suggesting that SAUSA300_*2383* is only transcribed in niches/media lacking glucose.

Since arginine transporters encoded by several bacterial species exhibit regulation via the ArgR family of regulators, we sought to determine whether SAUSA300_*2383* was regulated by AhrC (31–35). First, we found that SAUSA300_*2383* transcript was not significantly altered in an *ahrC* mutant during growth in CDMG, suggesting that CcpA is epistatic to regulation by AhrC (Fig. 7B). However, during growth in CDM where CcpA regulation is alleviated due to the lack of glucose, SAUSA300_*2383* transcript levels were higher (although not significant) in JE2 ∆*ahrC* as compared to JE2 (Fig. 7B). However, once arginine concentrations in CDM were reduced to 5.7 µM, SAUSA300_*2383* transcript was significantly reduced, in an *ahrC-*dependent manner, as compared to CDM. Similar transcriptional results were noted between growth in CDM vs CDM-P, suggesting that SAUSA300_*2383* is not specifically induced due to growth in medium lacking proline.

### SAUSA300_2383 transports arginine

**Fig 8:**
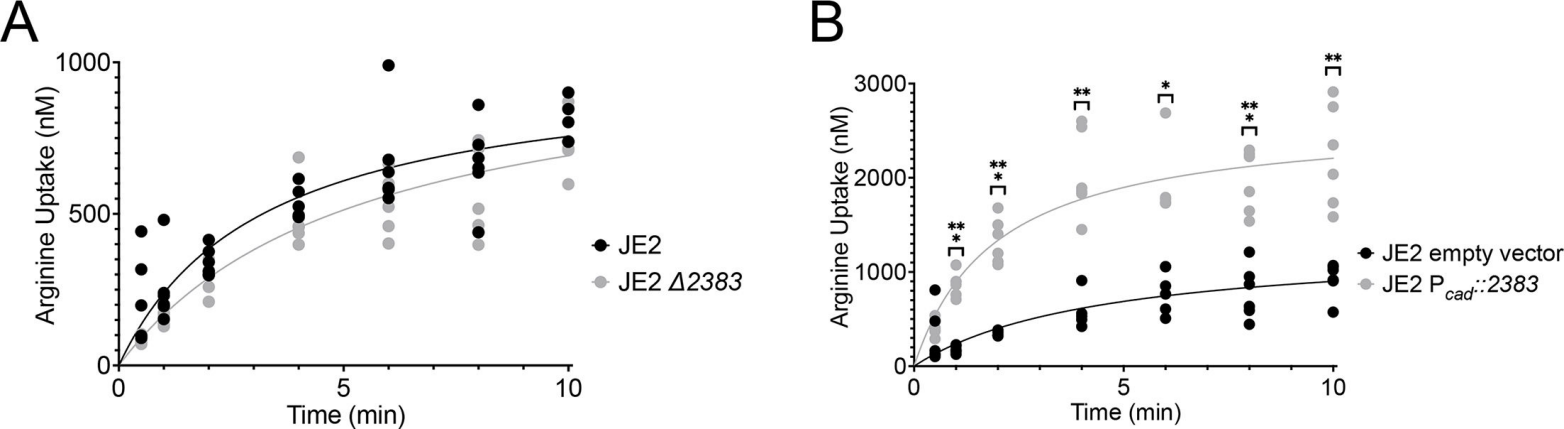
ArgT functions as an arginine transporter in *S. aureus*.^3^H-arginine transport assays measured the amount of radiolabeled arginine transported by *S. aureus* in CDM over a 10-minute period. There was a decrease, but not significantly significant, in arginine transport when JE2 ∆*2383* was compared to JE2 (**A**). However, the overexpression of SAUSA300*_2383* (JE2 P_cad_::*2383*) exhibited over a twofold increase in transport activity compared to JE2 empty vector when grown in 1 µg/mL chloramphenicol and 100 nM cadmium chloride (**B**). To account for experimental variability, all strains were normalized to JE2 and analyzed by non-linear regression using a first-order equation, based on technical duplicates from three independent experiments. Statistical significance was assessed via a mixed-effects analysis with Bonferroni test. *, *P* < 0.05; **, *P* < 0.01.

Our growth analyses indicate that SAUSA300_*2383* is critical for arginine transport during growth in media lacking proline. To confirm that the gene encodes an arginine transporter, we performed radiolabeled transport assays. JE2 and JE2 ∆*2383* were grown in CDM, washed, and placed in CDM lacking arginine (CDM-R) containing ^3^H L-arginine. A slight decrease in arginine transport was noted in JE2 ∆*2383* (Fig. 8A); however, as noted in Fig. 4B and C, other arginine transporters are expressed during growth in CDM. Furthermore, when SAUSA300_*2383* was overexpressed using the cadmium-inducible pBK123 vector (JE2 P_cad_:*2383*), we observed twice the amount of arginine transport as compared to vector alone (Fig. 8B). Based on these data, SAUSA300_*2383* was renamed ArgT for Arginine Transporter.

## DISCUSSION

These studies were designed to identify arginine transporters due to their proposed importance for *S. aureus* infection. Using the toxic arginine analog canavanine, we identified canavanine-resistant mutants with various SNPs in a proposed amino acid permease SAUSA300_*2383*. We subsequently have renamed this permease ArgT. Although our goal was to identify transporters that are expressed during growth in media with glucose and thus potentially expressed during the initial stages of infection, we instead identified ArgT, which is transcriptionally repressed during growth in media containing glucose via CcpA. Based on our studies, ArgT is expressed, along with other unidentified arginine transporters, in medium lacking glucose. However, it is required for arginine transport in medium lacking proline. Why is arginine transport dependent upon ArgT in medium lacking proline? We are unaware of other studies assessing a specific prokaryotic response that is induced due to a lack of proline. Transcriptional analysis revealed that a lack of proline does not alter *argT* transcription (Fig. 7B); however, it is possible that other, yet identified, arginine transporters are repressed via second messenger signaling due to a depleted intracellular proline. Laboratory studies have found that arginine is the primary substrate for proline biosynthesis in *S. aureus* via RocF/RocD/ProC (Fig. 1) and, in addition, it is becoming increasingly recognized that proline functions as a primary carbon source during *S. aureus* infection (36, 37). Further, proline is a critical osmolyte that is required for growth in media containing high salt conditions (38, 39). Indeed, recent studies have shown that growth in high salt conditions is more dependent upon proline biosynthesis via arginine than proline transport (37). More work is needed to understand why arginine transport in *S. aureus* is dependent upon ArgT in medium lacking proline. However, in hindsight, we would not have identified ArgT in our original agar-based canavanine screen if we had not used CDM-agarose lacking proline (CDM-PRE with peptides).

The canonical arginine biosynthesis repressor AhrC (ArgR family of repressors) forms a hexamer that binds to L-arginine and functions as a transcriptional regulator binding to ARG boxes upstream of arginine synthesis/transport genes (19). Regulation of arginine transporters via the ArgR family of regulators has been shown in *Streptococcus pneumoniae*, *Bacillus subtilis*, and *Escherichia coli*. In *S. pneumoniae*, AhrC and ArgR1 regulate arginine transporters in an arginine-dependent manner (31). When arginine concentrations are reduced to levels that impair growth, the transport of arginine is upregulated compared to conditions with excess arginine. Additionally, both AhrC and ArgR1 are needed to sense the amount of arginine and therefore bind and repress the promoters of arginine transporter genes (*abpA*, *artP*, *aapA*, and *abpB*) (31). In *B. subtilis*, AhrC represses arginine synthesis and activates its catabolism by binding to the ARG box containing promoter region of the *roc* operon (34, 40). Once the co-activator binds to the *roc* ARG box, there is an activation of transcription for the RocE arginine transporter (35). More is known about the regulation of *E. coli*-encoded arginine transporters. In the presence of arginine, ArgR binds and inhibits two major arginine transporters (*artPIQM-artJ* and *argT-hisJQMP*) as shown via binding assays and footprinting experiments (32, 41). Although the mechanism is not known, our studies revealed that AhrC represses argT when the arginine concentration is reduced in the medium (Fig. 7B). However, it is unclear how AhrC represses *argT* transcription in the absence of arginine. Nevertheless, these data suggest that ArgT is a low-affinity transporter as transcription is reduced as arginine concentrations become depleted intracellularly. Further, an *ahrC* mutation facilitated growth of JE2 *argT* in CDM-P, suggesting that other transporters can be identified by assessing the AhrC regulon. Moreover, although growth of JE2 *argT* is reduced in CDM-PE (Fig. 4A), growth is still observed, suggesting that another arginine transporter is active during these growth conditions. Therefore, similar to other nutrient acquisition systems in *S. aureus* (37), the pathogen encodes a repertoire of transporters that are differentially regulated, and additional studies will be designed to identify all arginine transporters in *S. aureus* due to their hypothesized importance during infection  

## MATERIALS AND METHODS

### Bacterial strains and culture conditions

Bacterial strains utilized in this study are listed in Table S1. *Bursa aurealis* transposon mutants were acquired from the NTML and backcrossed to *S. aureus* JE2 using Φ11 phage (42). Bacterial strains with multiple *Tn* insertions utilized various antibiotic markers as previously described (43). For growth analyses, overnight starter cultures were grown in 5 mL tryptic soy broth (TSB) at 37°C shaking at 250 rpm. Cultures were then washed with 1× phosphate-buffered saline (PBS) twice and inoculated to an optical density OD_600_ of 0.05. The bacterial cells were then added to a 96-well plate with CDM with (CDMG) or without (CDM) 3.5 mM glucose. Growth was measured in an Infinite 200 Pro device (Tecan) at 37°C, shaking at 250 rpm over a 24-hour period (aerobic growth; acetate is catabolized facilitating post-exponential growth using these growth conditions).

### Construction of JE2 **∆***2383* (*argT*)

A 1,000 bp region upstream and downstream of SASUA300*_2383* was amplified via PCR using primers 56–59 (Table S2) and cloned into pJB38, which was digested with EcoRI and XmaI. After Gibson Assembly (New England Biolabs) primers, JBLCL10F and JBLCL10R were used to confirm the integration of the PCR products. The plasmid was subsequently electroporated into *S. aureus* RN4220 (44), and Φ11 phage was utilized to transduce the plasmid into JE2 (45). Whole plasmid sequencing (Eurofins Genomics) confirmed the plasmid sequence. Allelic replacement was performed as previously described and confirmed using primers 39 and 44 (43).

### Construction of JE2 P_cad_*:2383*

SAUS300_*2383* was amplified utilizing Primers 110 and 111 (Table S2) and was cloned into pBK123 via Gibson Assembly (New England Biolabs). Once the plasmid was transformed into *E. coli* DH5α, PCR was used to confirm an accurate assembly (Primers oLH110 and oLH111). The plasmid was introduced into *S. aureus* RN4220 via electroporation (44) and was confirmed by PCR using primers oLH110 and oLH111. Finally, the plasmid was transduced into JE2 and JE2 ∆*2383* utilizing phage Φ11 (45). Eurofins Genomics Whole Genome Sequencing was used to verify the plasmid sequence. 1 µg/mL chloramphenicol and 100 nM cadmium chloride were utilized for selection and promoter induction, respectively.

### Toxic analog screen

Bacteria were grown overnight in TSB at 37°C shaking at 250 rpm. Once bacteria were pelleted by centrifugation, they were washed with 1× PBS and brought to an OD_600_ of 1. Strains were inoculated onto CDM lacking proline, arginine, and glutamate (CDM-PRE) but containing 0.1% trypticase peptones and 0.8% agarose with a cotton swab. Canavanine was added (2 mg) to the center disk, and plates were incubated at 37°C overnight. The zone of inhibition was measured from the edge of the canavanine disk to a full lawn of bacterial growth. Suppressor mutants that grew within the zone of inhibition were subsequently plated to verify resistance to canavanine.

### Whole genome sequencing

Genomic DNA was isolated from JE2, JE2 *arcD1 arcD2*, JE2 *arcD1 arcD2* suppressor mutants, JE2 ∆*2383*, and JE2 ∆*2383* suppressor mutants. First, bacteria were cultured overnight in 5 mL TSB at 37°C shaking at 250 rpm. The following day, 500 µL of the culture was centrifuged and was resuspended in 50 mM EDTA and lysostaphin (10 mg/mL stock). Samples were then processed with the Wizard Genomic DNA Purification Kit (Promega). Samples were measured via the Qubit (Thermo Fisher Scientific) to ensure 1 ng of DNA was used for sequencing. The Nextera XT DNA library preparation kit was used to prep genomic DNA for whole-genome sequencing. Samples were fragmented and tagged with unique indices before undergoing clean-up via AMPure XP beads (Beckman Coulter). This allowed for amplicons with greater than 500 bp to be selected for sequencing. Samples were run on an agarose gel to verify the presence of the library and the size of the amplicons. The library was then normalized to a concentration of 2 nm and pooled in preparation for sequencing. Since *S. aureus* is an AT-rich organism, PhiX was added at 15% to provide a diverse nucleotide composition, increasing the accuracy of base calling. The pooled samples were loaded into a MiSeq Reagent Kit V3 600 cycles. The MiSeq system then generated fastq files that were analyzed via the Qiagen CLC Workbench. Samples were aligned to the reference JE2 genome in the National Center for Biotechnology Information (CP020619).

### qRT-PCR transcriptional analysis

Starter cultures of JE2, JE2 ∆*ahrC*, and JE2 *ccpA::tetL* were grown overnight in 5 mL TSB in 37°C shaking at 250 rpm. Bacteria were then inoculated to an OD_600_ of 0.05 in the 50 mL of various medium conditions (CDMG, CDMG + 57 µM arginine, CDM, CDM + 5.7 µM arginine, and CDM-P) in a 500 mL flask. Flasks were incubated at 37°C shaking at 250 rpm until they reached mid-exponential phase (OD of 0.4–0.8). In a 15 mL falcon tube, 3 mL of cell culture was collected with 6 mL Qiagen RNAprotect Bacteria Reagent. Samples were briefly vortexed and incubated at room temperature for 5 minutes before they were centrifuged for 10 minutes at 5,000 rpm. The supernatant was then discarded, and tubes were inverted to ensure all liquid was removed before storing at −20°C for up to 2 weeks. Once the pellets were thawed, cells were resuspended in 150 µL RNase-free water and transferred to a microcentrifuge tube. Cells were centrifuged at full speed for 3 minutes, and the supernatant was discarded. Lysis buffer, containing 184 µL of water and 1 µL of 10 mg/mL lysostaphin, was added to the cells with 15 µL proteinase K. After a brief vortex, samples were incubated on a nutating platform shaker at room temperature for 10 minutes. Next, 700 µL of RLT buffer (containing 10 µL β-mercaptoethanol/1 mL RLT) was added to the samples and briefly vortexed. The suspension was transferred to tubes containing silica beads and processed in a bead beater at a speed of 6.0 for 45 seconds. After centrifuging samples for 10 seconds, 760 µL of supernatant was transferred to a new tube and mixed with 590 µL of 80% ethanol. Samples were mixed by pipetting then processed through the Qiagen RNeasy Kit for RNA isolation. In the reverse transcriptase reaction, 1 pg to 5 µg of RNA was utilized for cDNA synthesis. On ice, 10 µL reactions containing ezDNase enzyme and buffer were incubated in the thermocycler for 5 minutes at 37°C to remove residual genomic DNA. The samples were then processed through the SuperScript IV VILO Master Mix kit. The no-RT controls were checked via PCR to ensure no genomic DNA contamination. All primers and probes are listed in Tables S2 and S3. Primer/probe master mixes (20×) contained 10 µM primers and 4 µM probes. Each well of the qRT-PCR reaction contained 10 µL Taqman Fast Advanced Master Mix (Thermo Fisher), 1 µL 20× primer/probe master mix, 5 µL cDNA template, and 4 µL water. Each standard and cDNA sample was duplicated and ran on the QuantStudio 3 instrument (Thermo Fisher Scientific). To determine transcript copy number, dilutions of a plasmid containing the gene of interest (*2383*) were plotted as a standard curve. The data were then normalized to the curve and compared to the reference gene, *gyrB*, to determine the relative expression of SAUSA300_*2383* transcript.

### Arginine transport assay

*S. aureus* was grown in 5 mL of TSB overnight at 37°C shaking at 250 rpm. Bacteria were then washed in 1× PBS and resuspended to an OD_600_ of 0.05 in 50 mL of CDM in a 500 mL flask (10:1 flask to volume ratio). Once cells reached mid-exponential phase (OD_600_ = 0.4–0.8), they were concentrated to an OD_600_ of 10 in CDM-R. One-hundred microliters of cellular aliquots was placed on ice, while the 250 µM stock pot and CDM-R aliquots were kept in a 37°C water bath. The stock pot consisted of 25% ^3^H L-Arginine (Revvity) and 75% L-arginine. Cells were then diluted 1:10 in pre-warmed CDM-R, and 100 µL was added to a filter and then washed with 10 mL 1× PBS + 25 mM L-arginine as a control. The pre-warmed stock pot was diluted 1:10 into 900 µL of bacteria in CDM-R. At various time intervals (0.5, 1, 2, 4, 6, 8, and 10 minutes), 100 µL of sample was washed with PBS and 25 mM cold arginine through a nitrocellulose filter. After the filters were dried, Filter Count scintillation fluid (Revvity) was added, and once filters were dissolved (roughly 24 hours), samples were analyzed using the Perkin Elmer Tri-Carb 2910TR Scintillation counter. CPM was used to determine the amount of radiolabeled arginine that entered the cell at various time points. Transport assay curves were analyzed using non-linear regression and fitted to a first-order equation.

## SUPPLEMENTAL MATERIAL

Supplemental figures and tables jb.00362-25-s0001.docx Figures S1 and S2; Tables S1 to S3.

ASM does not own the copyrights to Supplemental Material that may be linked to, or accessed through, an article. The authors have granted ASM a non-exclusive, world-wide license to publish the Supplemental Material files. Please contact the corresponding author directly for reuse.
