# Supplementary material for: ArgT functions as an arginine transporter in Staphylococcus aureus
Source: J Bacteriol. 2025 Nov 28;207(12):e00362-25. doi: 10.1128/jb.00362-25 (PMC12713382; doi:10.1128/jb.00362-25)
Supplement: Supplemental figures and tables — Figures S1 and S2; Tables S1 to S3. [file jb.00362-25-s0001.docx]

**ArgT Functions as an Arginine Transporter in *Staphylococcus aureus***

Gabrielle F. Schulze, Itidal Reslane, Fareha Razvi, Luke D. Handke, McKenzie K. Lehman, and Paul D. Fey*

University of Nebraska Medical Center

Department of Pathology, Microbiology, and Immunology

Center for Staphylococcal Research

Omaha, NE. 68198

Supplementary Material


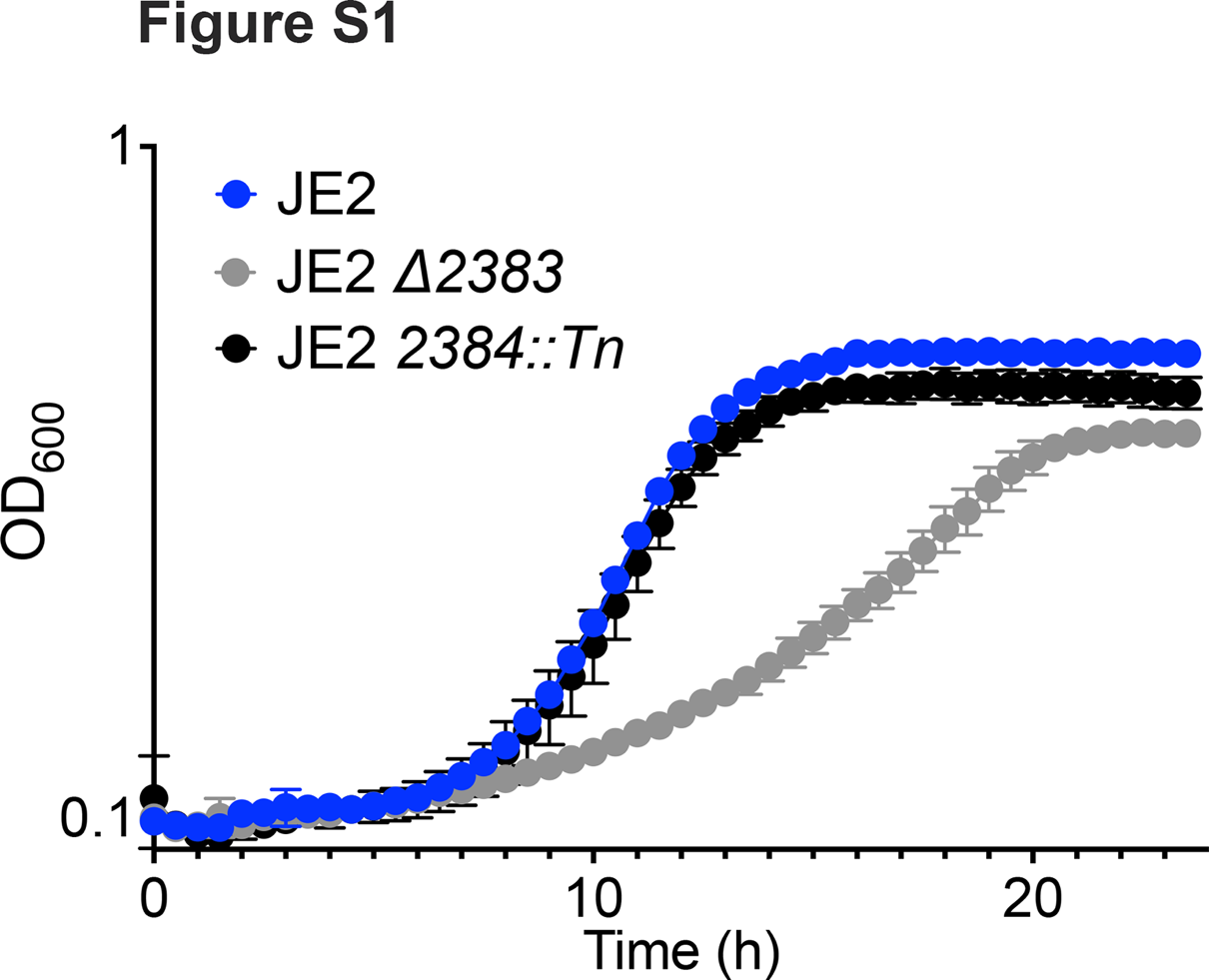


**Figure S1. SAUSA300_2384 Displays No Growth Defect in CDM-PE.** Growth analysis of JE2, JE2 ∆*2383,* and JE2 *2384::Tn* reveals SAUSA300_2384 does not display a phenotype in comparison to JE2 ∆*2383*. Data are represented by the mean +/- SD (n=3).


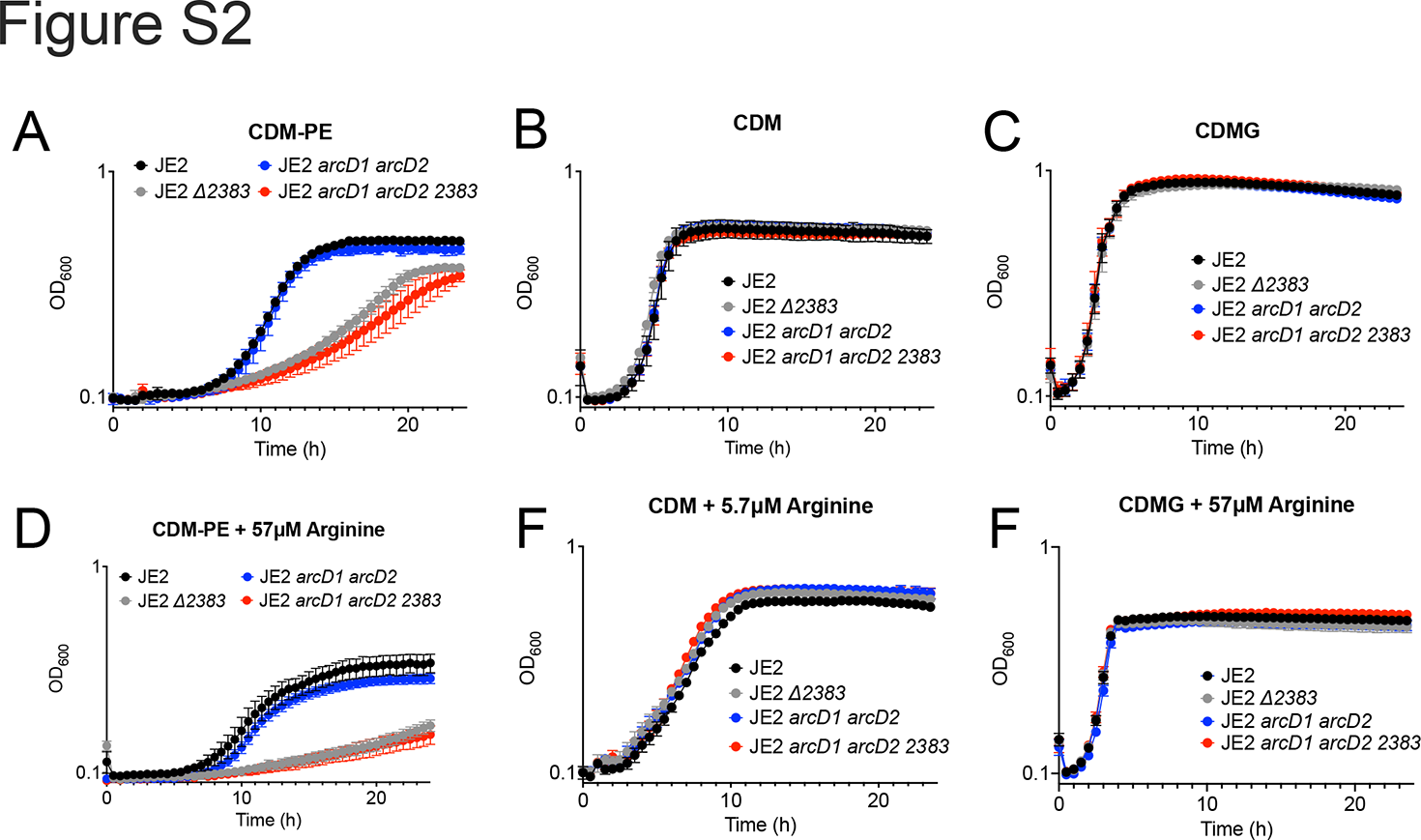


**Figure S2. ArcD1 and ArcD2 are not the Primary Arginine Transporters in Conditions Tested.** Growth analysis was performed evaluating JE2, JE2 ∆*2383*, JE2 *arcD1 arcD2*, and JE2 *arcD1 arcD2 2383* in various medium conditions **(A-F)**. JE2 *arcD1 arcD2* did not exhibit ay growth defects compared to JE2 in any of the conditions tested. Additionally, JE2 *arcD1 arcD2 2383* did not further reduce the growth rate of JE2 ∆*2383* indicating ArcD1 and ArcD2 are not the primary transporters of arginine in these medium conditions. Data (A-F) are represented by the mean +/- SD (n=3).

**Table S1: Bacterial strains and plasmids used in study**

| **Bacterial Strains/Plasmids** | **Relevant Phenotype** | **Source** |
| --- | --- | --- |
| pBK123 (empty vector) | Chloramphenicol-resistant derivative of shuttle plasmid pCN51 | [1] [2] |
| pJB38 | pJB33 with XhoI site removed | [3] |
| *S. aureus* JE2 | Wild-type, USA300 LAC cured of all three plasmids | [4] |
| *S. aureus* RN4220 | Restriction deficient NCTC8325-4 | [5] |
| *S. aureus* JE2 ∆*ahrC* | Markerless deletion in SAUS300_1469, arginine repressor | [6] |
| *S. aureus* JE2 ∆*2383* | Markerless deletion in SAUSA300_2383, arginine transporter | This study |
| *S. aureus* JE2 *arcD1::tet arcD2::spec* | Allelic replacement of *arcD1::Tn* (SAUSA300_2568) with tetracycline resistance cassette. Allelic replacement of *arcD2::Tn* (SAUSA300_0064) with spectinomycin resistance cassette. JE2 *arcD2::spec* transduced into JE2 *arcD1::tet* via Φ11 | This study |
| *S. aureus* JE2 *arcD1::tet arcD2::spec 2383::Tn* | SAUSA300_2383, *2383::Tn* transduced into JE2 *arcD1::tet arcD2::spec* | This study |
| *S. aureus* JE2 ∆*ahrC 2383::Tn* | SAUSA300_2383, *2383::Tn* transduced into JE2 ∆*ahrC* via Φ11 | This study |
| *S. aureus* JE2 *2384::Tn* | SAUSA300_2384, *2384::Tn* transduced into JE2 | This study |
| *S. aureus* JE2 P_cad_:: *2383* | pBK123 with the *2383* ORF cloned behind the cadmium-inducible promoter at the BamHI/EcoRI sites. Transduced into JE2 via Φ11 | This study |
| *S. aureus* JE2 ∆*2383* P*_cad_:: 2383* | pBK123 with the *2383* ORF cloned behind the cadmium-inducible promoter at the BamHI/EcoRI sites. Transduced into JE2 ∆*2383* via Φ11 | This study |
| *S. aureus* JE2 *ccpA::tetL* | Allelic replacement mutation in *ccpA*; transduced from MST14: Tet^r^ | [7] |

1. Sharma-Kuinkel, B.K., et al., *The Staphylococcus aureus LytSR two-component regulatory system affects biofilm formation.* J Bacteriol, 2009. **191**(15): p. 4767-75.

2. Charpentier, E., et al., *Novel cassette-based shuttle vector system for gram-positive bacteria.* Appl Environ Microbiol, 2004. **70**(10): p. 6076-85.

3. Bose, J.L., P.D. Fey, and K.W. Bayles, *Genetic tools to enhance the study of gene function and regulation in Staphylococcus aureus.* Appl Environ Microbiol, 2013. **79**(7): p. 2218-24.

4. Fey, P.D., et al., *A genetic resource for rapid and comprehensive phenotype screening of nonessential Staphylococcus aureus genes.* mBio, 2013. **4**(1): p. e00537-12.

5. Kreiswirth, B.N., et al., *The toxic shock syndrome exotoxin structural gene is not detectably transmitted by a prophage.* Nature, 1983. **305**(5936): p. 709-12.

6. Reslane, I., et al., *Catabolic Ornithine Carbamoyltransferase Activity Facilitates Growth of Staphylococcus aureus in Defined Medium Lacking Glucose and Arginine*. 2022, American Society for Microbiology.

7. Nuxoll, A.S., et al., *CcpA Regulates Arginine Biosynthesis in Staphylococcus aureus through Repression of Proline Catabolism*. 2012, Public Library of Science (PLoS).

**Table S2. Primers used in study.**

| **Name** | **Sequence** | **Comments** |
| --- | --- | --- |
| Primer 56 | ATCACGAGGCCCTTTCGTCTTCAAGCTATCACACTTTTACGAAAATGAACATGGCC | Forward Primer used for fragment 1 of *SAUSA300_2383* (Gibson assembly) |
| Primer 57 | GAGATGTTTAAATCAATTTTCTTGCCATAATTTTCCTCCTCAGG | Reverse Primer used for fragment 1 of *SAUSA300_2383* (Gibson assembly) |
| Primer 58 | GCAAGAAAATTGATTTAAACATCTCGCTCTAGTATCAGTTCAACC | Forward Primer used for fragment 2 of *SAUSA300_2383* (Gibson assembly) |
| Primer 59 | CCTGCAGGTCGACTCTAGAGGATCCCAAATACAAAACCATTTAAGGCATAACTAAATGTGTTCC | Reverse Primer used for fragment 2 of *SAUSA300_2383* (Gibson assembly) |
| JBLCL10F | CCCGAAAAGTGCCACCTGACGTC | Forward primer used to verify SAUSA300_2383 allelic replacement plasmid |
| JBLCL10R | CGAAAATGCCTCACATTTGTGCCACC | Reverse primer used to verify SAUSA300_2383 allelic replacement plasmid |
| Primer 39 | AAGTAGTGGCGCGATAACGG | Forward primer used to confirm JE2 2383 allelic replacement |
| Primer 44 | GGCGATTGTCCCTATCTTCATACC | Reverse primer used to confirm JE2 2383 allelic replacement |
| Primer 110 | AGGTCGACTCTAGAGGATCCAGGAGGAAAATTATGGCAAGAAAATTGCA | Forward primer used to amplify SAUSA300_2383 for complementation |
| Primer 111 | CATTAGAATAGGCGCGCCTGTTATTGATTGTCATATCTGAAATGACCGATGTCA | Reverse primer used to amplify SAUSA300_2383 for complementation |
| oLH110 | AGAAAAGAAGGAAAACTAGC | pBK123 forward to confirm SAUSA300_*2383* cloning |
| oLH111 | CAAAATTATACATGTCAACG | pBK123 reverse to confirm SAUSA300_*2383* cloning |
| oLH17 | CAAATGATCACAGCATTTGGTACAG | *gyrB* Forward used for qRT-PCR |
| oLH18 | CGGCATCAGTCATAATGACGAT | *gyrB* Reverse used for qRT-PCR |
| Primer 77 | GTCGTTAACTTTGTCGTGCTAAC | SAUSA300_*2383* Forward used for qRT-PCR |
| Primer 79 | TAACCCTTGTTGTGACAGTCC | SAUSA300_*2383* Reverse used for qRT-PCR |

**Table S3. Probes used in study**

| **Name** | **Sequence** | **5’ Modification** | **3’ Modification** | **Comments** |
| --- | --- | --- | --- | --- |
| *2383* | ACCACTATTTGTAGCAGAGGCCGC | HEX | BHQ1 | Taqman probe used for detection of *2383* transcript in qRT-PCR |
| *gyrB* | AATCGGTGGCGACTTTGATCTAGCGAAAG | 6-FAM | BHQ1 | Taqman probe used for detection of *gyrB* transcript in qRT-PCR |
